# Novel biogenic silver nanoparticles produced by *Enterobacter xiangfangensis* Pb204 reinstate the activity of specific antibiotics against resistant ESKAPE pathogens

**DOI:** 10.1039/d5na00787a

**Published:** 2025-12-15

**Authors:** Prabhavathi Sathish Sundar, Rofhiwa Musoliwa, Kulsum Kondiah

**Affiliations:** a Department of Biotechnology and Food Technology, Faculty of Sciences, University of Johannesburg South Africa psundar@uj.ac.za 216030804@student.uj.ac.za kulsumk@uj.ac.za

## Abstract

Antimicrobial resistance (AMR) in pathogenic bacteria remains a major challenge and critical threat to the global healthcare industry, demanding alternative therapeutic strategies. Among the various nanomaterials studied, silver nanoparticles (Ag-NPs) have shown promising antibacterial properties due to their broad-spectrum activity, oligodynamic effect, and reduced possibility of inducing microbial resistance. This study investigates the antimicrobial efficacy of biogenically synthesised silver nanoparticles using a cell-free extract of *Enterobacter xiangfangensis* Pb204 combined with antibiotics against eight pathogenic bacterial strains, including ESKAPE pathogens (*Enterococcus faecium*, *Staphylococcus aureus*, *Klebsiella pneumoniae*, *Acinetobacter baumannii*, *Pseudomonas aeruginosa*, *Enterobacter* spp.), *E. coli*, *and Vibrio cholerae.* The biogenic Ag-NPs were characterised by ultraviolet-visible (UV-Vis) spectroscopy and transmission electron microscopy (TEM) with energy-dispersive spectroscopy (EDS) analysis. Disc diffusion assays demonstrated that biogenic Ag-NPs (21 µg and 25 µg) effectively inhibited the growth of all tested pathogens. When Ag-NPs were combined with antibiotics amoxicillin/clavulanic acid (AMC), ampicillin (AMP), ciprofloxacin (CIP), meropenem (MEM), and vancomycin (VAN), most inhibition zones expanded, with the greatest synergistic effect observed in combination with vancomycin against *Enterococcus faecium*. These results support the potential of combined therapies using antibiotics and biogenic Ag-NPs to combat the effects of AMR in clinically significant pathogens.

## Abstract 2

Synthesis and antibacterial activity of Ag-NPs by *Enterobacter xiangfangensis* Pb204 which was previously isolated from mining effluent. The biosynthesized Ag-NPs exhibited a synergistic antimicrobial effect when combined with antibiotics.

## Graphical abstract

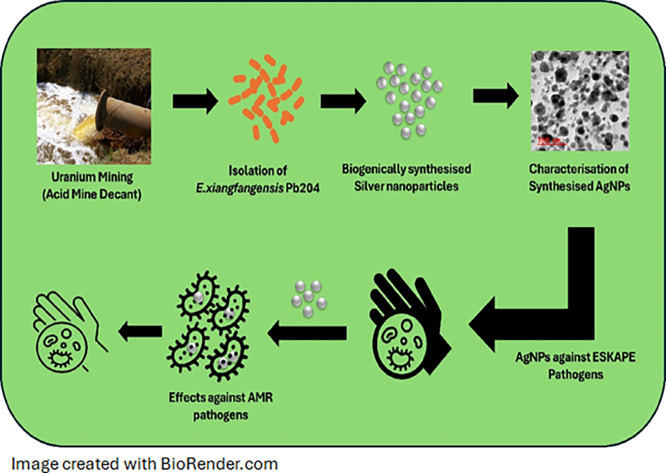

## 1. Introduction

The increasing number of infections caused by multidrug-resistant bacteria is considered a major challenge to public health. The Infectious Diseases Society of America (IDSA) has named a group of drug-resistant bacteria, known as “ESKAPE” pathogens, as they can survive the biocidal action of antibacterial drugs. The ESKAPE pathogens are *Enterococcus faecium*, *Staphylococcus aureus*, *Klebsiella pneumoniae*, *Acinetobacter baumannii*, *Pseudomonas aeruginosa*, and *Enterobacter* spp. These pathogens are also known as extensively drug-resistant or multidrug-resistant (MDR) bacteria. They are responsible for Hospital Acquired Infections (HAIs), including the majority of nosocomial infections, and are considered a serious threat to patients with compromised immune systems. The World Health Organization (WHO) has also listed ESKAPE as part of the 12 bacteria that urgently require new antibiotics.^1^ Various water sources, soil, food, plants, and household waste are common environmental reservoirs for ESKAPE pathogens, which contribute to the development of antimicrobial resistance.^2^ The development of resistance to antibiotics by pathogenic bacteria has presented a significant challenge to the advancement of antimicrobial drugs. This has led to the resurgence of the use of silver as an antimicrobial agent.^3^

Recent advances in nanotechnology have drawn researchers to apply metal nanoparticles as an effective method for controlling specific pathogenic microorganisms that cause infectious diseases. Among the nano systems, silver nanoparticles (Ag-NPs) are presently viewed as an alternative antibacterial agent with the ability to fight the emergence and reemergence of bacterial MDR.^4^ These Ag-NPs possess significant biological properties, as they are effective antimicrobial agents with multiple modes of action against a wide range of bacteria, including antibiotic-resistant strains.^5^ Additionally, Ag-NPs have been shown to be more effective in their antimicrobial activities against viruses, fungi, and other eukaryotic microorganisms. For example, anti-fungal activity has been reported against various pathogenic fungi, including *Candida albicans*, *Aspergillus fumigates*, *A. flavus*, *A. niger*, *Trichophyton rubrum*, and *Penicillium species*.^6^ Replication of HIV-1 virus was reported to be inhibited by Ag-NPs ranging from 5–20 nm, providing evidence of their antiviral activity.^7^ Silver nanoparticles exhibit potent and broad-spectrum antibacterial activity through multiple complementary mechanisms. Firstly, Ag-NPs adhere to bacterial cell walls and membranes, causing structural damage and increased permeability, which leads to cell lysis and leakage of intracellular contents.^8^ Once inside the cell, Ag-NPs induce the production of reactive oxygen species (ROS), which cause oxidative stress and damage essential biomolecules, including DNA, proteins, and lipids.^9^ Additionally, Ag-NPs release silver ions (Ag^+^), which bind to thiol (–SH) groups in enzymes and proteins, disrupting key metabolic processes such as cellular respiration and replication.^10^ These multifaceted mechanisms not only enhance the bactericidal effect but also make it difficult for bacteria to develop resistance, positioning Ag-NPs as promising candidates against multidrug-resistant pathogens.^11^ Silver nanoparticles have been reported to kill a wide range of bacteria, including ampicillin-resistant *E. coli*, *P. aeruginosa*, vancomycin-resistant *S. aureus* (VRSA), erythromycin-resistant Streptococcus pyogenes, and methicillin-resistant *S. aureus* (MRSA).^8^ These antibacterial activities were also reported against bacterial pathogens such as *P. aeruginosa*, *K. pneumoniae*, *E. coli*, *S. aureus*, *E. faecium*, and *Enterobacter* sp., among others.^12,13^

Many researchers support the idea that use of Ag-NPs can be exploited in the field of medicine, dental materials, textiles fabrics, water treatment *etc.*, and possess low toxicity to human cells, low volatility and high thermal stability.^14–17^ The use of drug combinations, two or more antimicrobial drugs to combat MDR bacteria, is already employed in cancer therapy, malaria, and HIV patients.^18^ Novel emerging antimicrobials that combine Ag-NPs and antibiotic compounds are undergoing clinical evaluations.^2,19–22^ Moreover, the synergistic effect of Ag-NPs and antibiotics can enable the use of low concentrations of Ag-NPs with reduced toxicity to humans. Therefore, the combination of antibiotics with Ag-NPs is viable for enhancing the antibacterial efficacy of antibiotics compared to their activity alone when used in clinical settings.^23,24^ Ag-NPs synthesized using *H. sabdariffa* flower extract exhibited strong antibacterial activity, and their combination with fosfomycin showed enhanced efficacy against drug-resistant nosocomial pathogens.^25^

**Fig. 1:**
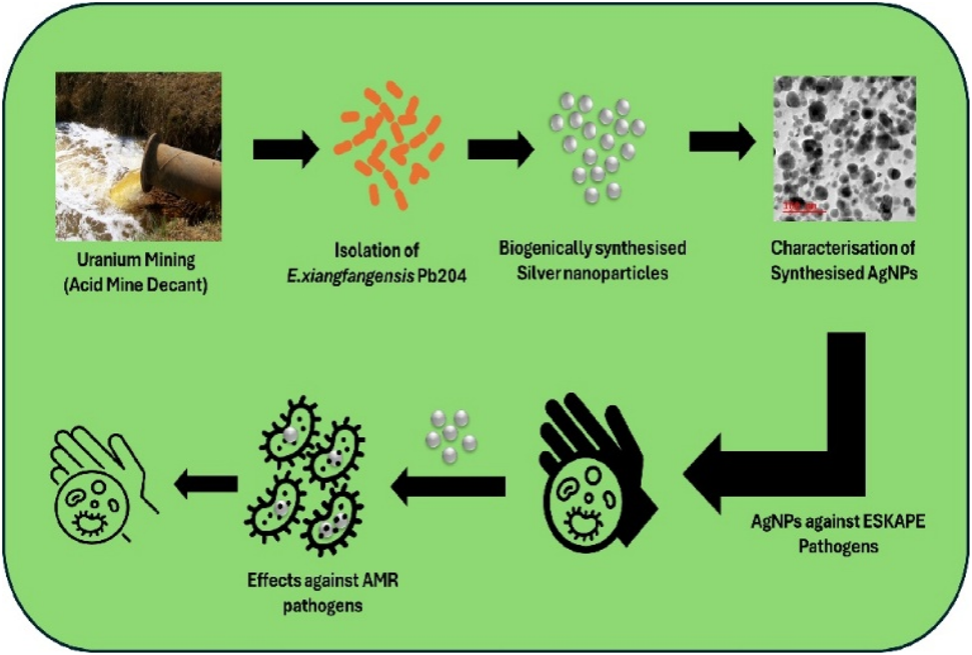
Green synthesis of silver nanoparticles from acid mine decant and its effects against AMR pathogens. Image created with BioRender.com.

*Enterobacter xiangfangensis*, a member of the *Enterobacter cloacae* complex, has emerged as a promising candidate for green nanotechnology due to its robust metal-reducing capabilities and adaptability. It demonstrates an inherent ability to tolerate and bio-reduce toxic metal ions, such as silver (Ag^+^), into stable nanoparticles *via* extracellular enzymatic mechanisms.^26^ The bacterium's metabolic versatility enables the production of biomolecules such as reductases, peptides, and extracellular polysaccharides, which act as both reducing and capping agents, facilitating the formation of well-stabilized Ag-NPs with uniform morphology.^27^ Additionally, *E. xiangfangensis* exhibits rapid growth, resilience under stress, and adaptability to diverse substrates, making it a cost-effective and scalable option for nanoparticle synthesis.^28^ Compared to commonly used strains like *Escherichia coli* or *Bacillus subtilis*, it offers superior nanoparticle yield and stability critical for biomedical applications, owing to its genome encoding stress response genes and metal ion transporters that enhance silver resistance and reduction capacity.^29^ These attributes collectively position *E. xiangfangensis* as an efficient and eco-friendly microbial platform for the synthesis of Ag-NPs. This study investigated the synergistic usage of biogenic Ag-NPs with classical antibiotics to help overcome the growing antimicrobial resistance (AMR) of ESKAPE pathogens, as shown in Fig. 1.

## 2. Experimental

### 2.1. Materials

Silver nitrate, acetone, sodium hydroxide, LB broth, and glycerol were purchased from Sigma Aldrich (Missouri, United States). Experiments were performed on five classical antibiotic discs: Vancomycin (30 µg), ciprofloxacin (5 µg), ampicillin (25 µg), meropenem (10 µg), and amoxicillin/clavulanic acid (20 µg/10 µg) (Mast Group Limited, Liverpool, U.K.).

### 2.2. Bacterial culture and growth conditions

Pure cultures of bacterial clinical isolates that included the ESKAPE pathogens *Enterococcus faecium*, *Staphylococcus aureus*, *Klebsiella pneumoniae*, *Acinetobacter baumannii*, *Pseudomonas aeruginosa*, *Escherichia coli*, *Enterobacter cloacae and Vibrio cholera* from Water and Health Research Centre, University of Johannesburg. *Enterobacter xiangfangensis* Pb204 was isolated from an acid mine decant-released uranium mine in South Africa (26°06 = 26.8S, 27°43 = 20.2E),^30^ and cultured by the Environmental Biotechnology Research Group, University of Johannesburg, South Africa.

### 2.3. Bacterial-based biosynthesis of silver nanoparticles (biogenic Ag-NPs)

*Enterobacter xiangfangensis* Pb204 was cultured in 5 mL of LB broth and incubated at 37 °C overnight. The pre-inoculum was further inoculated into LB broth 1 : 100 (v/v) and incubated at 37 °C for 24 h with shaking at 200 rpm. After incubation, the medium was centrifuged twice at 5000×*g* for 15 minutes at 25 °C in a Multifuge XIR (Thermo Fisher Scientific, Germany) to obtain supernatant. The cell-free extract was added to a 1 mM filter-sterilized AgNO_3_ solution at a final concentration of 100 mL (pH 7) and incubated at 120 rpm for 72 h at 37 °C. Silver nanoparticle production was visually observed during the incubation period, as evidenced by a change in the color of the reaction mixture. The nanoparticles were harvested from the reaction mixture using a centrifuge at 20 000×*g* for 15 minutes at 25 °C and then washed with 50% acetone to minimize any media interference. The wash step was repeated twice. The purified were dried overnight in a desiccator and maintained at 4 °C.

### 2.4. Characterization of biosynthesised silver nanoparticles

#### 2.4.1. Ultraviolet-visible (UV-Vis) spectroscopy

Biosynthesised silver nanoparticles initial confirmation was done using UV-Vis spectrophotometry. About 2 mL of the biosynthesized mixture taken from 0 h, 24 h, 48 h, and 72 h samples, was transferred into a quartz cuvette during the reaction period and the measurements were carried out at an operational range of wavelength scan between 300-800 nm in the kinetic BioSpectrometer® (Epperndorf, Germany).

#### 2.4.2. Transmission electron microscopy (TEM) with energy dispersive X-ray spectroscopy (EDX)

Transmission electron microscopy was employed to clarify the relative morphology, size, and nature of the nanoparticles. Biogenic Ag-Nps were prepared by drop-coating them onto the 200-mesh carbon-coated copper grid and allowed to dry at room temperature for 24 h. The sample was analyzed for size and morphology on JEOL 2100 electron microscope operating at a voltage of 200 Kv (HRTEM, JOEL Ltd, Tokyo, Japan). The size of the biogenic Ag-NPs and data analysis of the TEM images was accomplished using Image J software and the elemental analysis was carried by EDX Spectroscopy.

### 2.5. Disc diffusion assays

The disc diffusion method was employed to evaluate the antibacterial activity of biogenic Ag-NPs, antibiotics, and their combination against bacterial isolates. Bacterial cultures were grown in Mueller–Hinton (M–H) broth, standardised to 0.5 McFarland (1 × 10^8^ cells per mL), and spread on M–H agar plates. Antibiotic discs, Ag-NP-loaded discs (2.5, 5, 10,12 mM equivalent to 5, 11, 21, 25 µg), and control discs (20 µL sdH_2_O) were placed on the plates. For combination studies, 21 and 25 µg Ag-NPs were used with antibiotics. Plates were incubated at 37 °C for 24 h, and inhibition zones were measured. Antibacterial activity was compared to CLSI guidelines, and the percentage fold increase in inhibition zones was calculated (Fayaz *et al.*, 2010).^19^Fold increase in% = {(*X* − *Y*)/*Y*} × 100(Where: *Y* is the inhibition zone of antibiotic alone; *X* is the inhibition zone of biogenic Ag-NPs and antibiotics).

### 2.6. Statistical analysis

All measurements represent the mean diameter of the inhibition zones (in mm) ± standard error (SE), calculated from three independent experiments, each performed in triplicate (*n* = 3). Statistical analysis was conducted using one-way analysis of variance (ANOVA) in Minitab software.

## 3. Results and discussion

The emergence and reemergence of MDR bacterial pathogens have been a major concern to public health for over a decade.^31,32^ Their increasing resistance to antibiotics has placed great strain on the pharmaceutical industry to produce new or alternative treatments that can be effective against such pathogens. As a result, the present study sought to determine if antibiotic effectivity can be increased in the presence of biogenic Ag-NPs for the treatment of ESKAPE pathogens.

Biosynthesis of biogenic Ag-NPs from *E. xiangfangensis* Pb204 was confirmed due to the presence of a characteristic brown colour change, characteristic SPR absorption peak and visual observation of spherical nanoparticles using TEM. The change in the colour of reaction medium depicts the excitation of surface plasmon vibrations in the metal NPs (Ahmad *et al.*, 2003; Algebaly *et al.*, 2020) which occurs due to reduction of Ag^+^ to Ag^0^.^33,34^ These findings are similar to those reported in other studies wherein a yellow to brown colour of the reaction medium was formed due to the reduction of Ag^+^ to Ag^0^.^35–38^

### 3.1. Confirmation of biogenic silver nanoparticles (Ag-NPs) synthesis

**Fig. 2:**
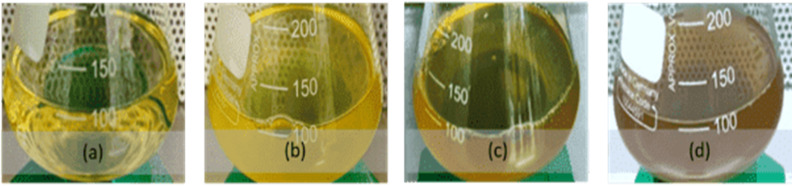
Change in the colour of reaction mixture over time due to reduction of Ag^+^ ions to Ag-NPs when cell extract of *Enterobacter xiangfangensis* Pb204 was incubated in the presence of 1 mM AgNO_3_: (a) 0 h, (b) 24 h, (c) 48 h and (d) 72 h.

The experimental solution was visually observed for a colour change that would indicate the reduction of Ag^+^ ions to Ag-NPs. The solution gradually changed from pale yellow (Fig. 2a) to dark brown over a reaction time of 72 h (Fig. 2d). This formed a preliminary indication of successful synthesis as a reaction mixture colour change to dark brown represents a primary indication of biogenic Ag-NPs formation. It was observed that the intensity of brown colour increased over time due to the ongoing bio-reduction of Ag^+^ ions into higher concentrations of biogenic Ag-NPs.

### 3.2. Characterisation of biogenic Ag-NPs

#### 3.2.1. UV-vis spectroscopy analysis

**Fig. 3:**
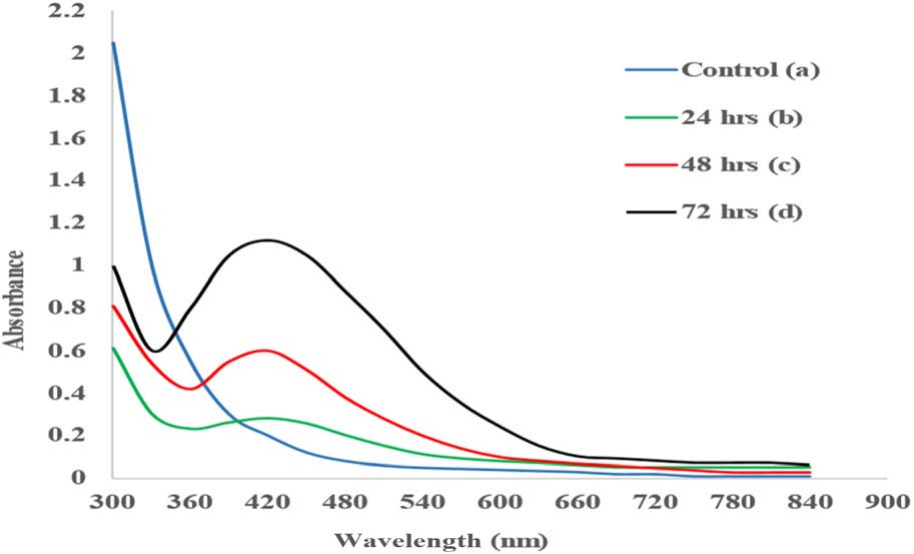
UV-Vis spectral profile of (a) control and biogenic Ag-NPs over time at (b) 24 h (c) 48 h and (d) 72 h.

It is well known that Ag-NPs possess a characteristic absorbance peak between a wavelength of 390 nm to 450 nm.^39^ In this study, the UV-Vis spectra were measured from 350 to 800 for samples collected at 24, 48 and 72 h. It was observed from the absorption spectrum that the surface plasmon resonance (SPR) band of biogenic Ag-NPs occurred at approximately 420 nm (Fig. 3b–d), while an absorption peak was not observed for the control reaction (Fig. 3a). It has been reported that the reduction of Ag^+^ to Ag^0^ corresponds to absorption at 420 nm.^40^ It is evident that the absorption steadily increases in intensity as a function of reaction time. Absorption peaks appear broad with a low intensity when the reaction time is shorter compared to longer reaction times.^33^

#### 3.2.2. Transmission electron microscopy with EDS

**Fig. 4:**
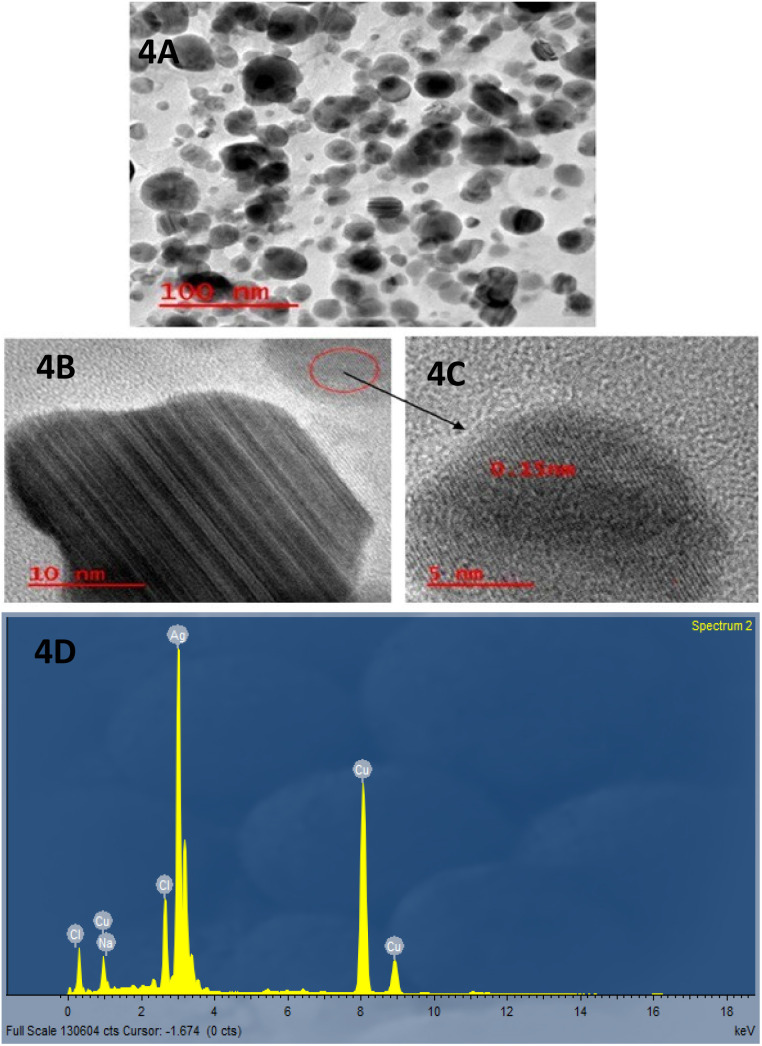
TEM image of biogenic Ag-NPs produced using the cell-free extract of *Enterobacter xiangfangensis* Pb204 in the presence of 1 mM AgNO_3_ (pH 7) at 37 °C for 24 h (captured in different scale bars, A: 100 nm, B: 10 nm, C: 5 nm), and D: EDX.

The TEM technique was used to further confirm the production of the extracellular biogenic Ag-NPs with clear geometric boundaries. Fig. 4A–C show the morphological structure of the biogenic Ag-NPs. TEM image reveals that biogenic Ag-NPs produced after 24 h were few spherical and few elongated particles and were confirmed to range between 12–40 nm, and predominantly there are two different sizes (23.09 ± 0.85; 36.67 ± 1.02) measured using ImageJ software. Moreover, the TEM images at a smaller scale in Fig. 4C showed the presence of a lattice fringe with an interplanar distance of 0.15 nm, indicating the crystalline nature of the NPs.

Energy dispersive X-ray spectroscopy further confirmed the presence of silver elements in the biogenic Ag-NPs (Fig. 4). The broadening of peaks is indicative of polydispersity of NPs in the medium, and the absence of additional absorption peaks at higher wavelengths is related to spherical or spherical-like shape uniformity of the NPs^22,41^ as confirmed by TEM. Longer reaction time resulted in larger particle size, ranging from 12–40 nm, and a tendency of NPs to form aggregates was observed. The EDS analysis revealed the presence of only metallic elements, primarily silver, without significant signals from non-metallic components, supporting the metallic nature of the synthesized nanoparticles. Although the exact mechanism of silver nanoparticle formation by *E. xiangfangensis* remains to be elucidated, similar biosynthetic pathways have been reported in other Gram-negative bacteria such as *Escherichia coli* and *Pseudomonas aeruginosa*, where enzymatic reduction of silver ions and the involvement of extracellular proteins have been proposed. These comparisons provide a preliminary framework to hypothesize potential mechanisms in *E. xiangfangensis*, which may involve similar reductase activity or secretion of electron-donating biomolecules.^33,42^ This is due to the secretion of protein components into the medium from the bacterial biomass, which may be the primary factor in reducing metal ions to NPs. Consequently, the proteins may also bind to the NPs, enhancing the stability of absorption.^43^

#### 3.2.3 Powder X-ray diffraction analysis

**Fig. 5:**
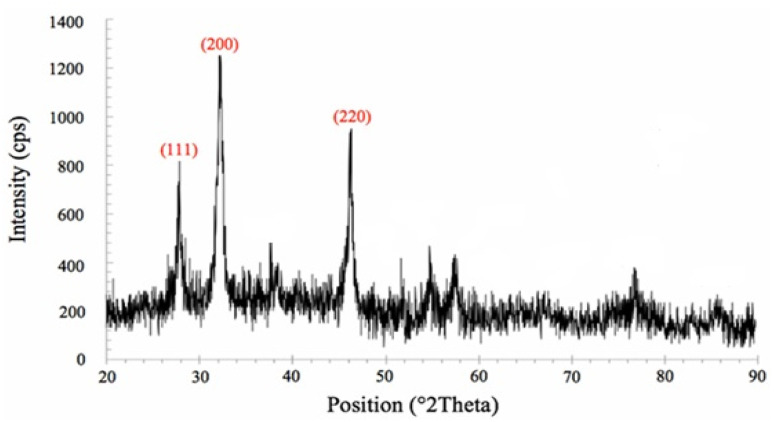
PXRD diffractogram of the lyophilized powder of biosynthesized Ag-NPs.

Fig. 5 shows the XRD pattern of the as-prepared Ag-NPs. Reflection peaks matched well with the standard reflection peaks of metallic silver in the {111}, {200}, and {220} planes of the fcc structure (JCPDS file: 65-2871). It is unclear whether metal alloys, individual metallic NPs, or combinations of these were formed. The broadening of peaks indicates the nano-sized nature of all NP samples.^44^ The biological synthesis of Ag-NPs is a common process, although its mechanism remains poorly understood.^45^ Using *B. subtilis*, the synthesis of polydisperse Ag NPs ranging from 20 nm to 60 nm was achieved without the addition of Cl-ions. The enzyme responsible for reducing Ag^+^ ions was hypothesized to be a membrane-bound 37 kDa nitrate reductase enzyme.^46^

### 3.3. Antibacterial assays

The standard antibiotics used in the study included AMC, AMP, CIP, MEM, and VAN. Bacterial resistance or sensitivity to antibiotics was interpreted according to the CLSI criteria guidelines.^47^ Where guidelines were unavailable, the results were interpreted in reference to the existing literature.

#### 3.3.1. Antibiotics disc diffusion assay

All bacterial strains used in the study were found to be resistant to at least two of the antibiotics tested, as illustrated in Table 1. In this study, CIP and MEM exhibited the strongest inhibitory activity against all bacterial pathogens except for *V. cholerae*, whose growth was inhibited the most by AMP. In particular, susceptibility (S) to MEM and CIP was recorded for *S. aureus*, *A. baumannii*, *P. aeruginosa*, *and E. cloacae* while intermediate (I) sensitivity was recorded for *K. pneumoniae* and *E. coli*. Breakpoint data for activity of MEM against *S. aureus* and *E. faecium* is not available;^47^ however, both strains were effectively inhibited by CIP. On the contrary, majority of the tested strains were resistant (R) to VAN where no zones of inhibition were observed except in the case of *S. aureus* and *E. faecium* with inhibition zones of 10.33 ± 2.89 and 7 ± 1 diameter, respectively. Antibiotic resistance to AMP and AMC was also recorded for all strains except *A. baumannii* and *V. cholera* for which large zones of inhibition were observed but no breakpoint data is available for classification of the antimicrobial profile.

**Table 1:** Inhibition zones for eight bacterial isolates. Data is demonstrated as mean ± S.D. (*n* = 3)

Pathogen	Mean ± S.D. Inhibition zone diameter values (mm)						
	Antibiotic^a^					Silver nanoparticles	
	AMC 20/10 µg	AMP 25 µg	CIP 5 µg	MEM 10 µg	VAN 30 µg	Ag-NPs 21 µg	Ag-NPs 25 µg
*E. faecium*	11.33 ± 0.58 (R)	15.33 ± 2.52 (R)	26.67 ± 1.15 (S)	23.67 ± 0.58	7 ± 1 (R)	12.33 ± 0.58	14.67 ± 1.53
*S. aureus*	14.33 ± 0.58 (R)	18.67 ± 1.15 (R)	27 ± 1 (S)	25.33 ± 0.58 (S)	10.33 ± 2.89	7.33 ± 0.58	10 0.33 ± 0.58
*K. pneumoniae*	11.33 ± 2.08 (R)	—	22.33 ± 2.08 (I)	21.33 ± 1.15 (I)	—	7.33 ± 0.58	9.67 ± 1.53
*A. baumannii*	15.33 ± 1.15	15.67 ± 1.15	29.67 ± 1.53 (S)	27.33 ± 0.58 (S)	—	7.33 ± 0.58	10.67 ± 0.58
*P. aeruginosa*	—	—	30.33 ± 0.58 (S)	26.33 ± 0.58 (S)	—	7.67 ± 0.58	11.33 ± 0.58
*E. cloacae*	12.67 ± 0.58 (R)	—	30.67 ± 1.15 (S)	26.67 ± 0.58 (S)	—	7.33 ± 0.58	11.33 ± 1.53
*E. coli*	8.33 ± 00.58 (R)	—	23.67 ± 1.53 (I)	21.67 ± 0.47 (I)	—	7.33 ± 0.58	10.67 ± 0.58
*V. cholerae*	13.33 ± 0.47	22.5 ± 0.71	15.67 ± 0.47	13.67 ± 0.47	—	7.67 ± 0.58	10.67 ± 0.58

a Bacterial isolates were interpreted as (−) no zone of inhibition, susceptible (S), intermediate (I), or resistant (R) according to CLSI M100 Performance Standards for Antimicrobial Susceptibility Testing (2021).

#### 3.3.2. Biogenic silver nanoparticles (Ag-NPs) disc diffusion assay

A disc diffusion assay was performed to determine which concentration (5, 11, 21, or 25 µg) of biogenic Ag-NPs would be effective at inhibiting the growth of the eight bacterial pathogens tested in the study. The average diameters of the inhibition zones for 21 and 25 µg concentrations are shown in Table 1. No inhibition was observed when 5 and 11 µg of Ag-NPs were used for all isolates tested.

#### 3.3.3. Biogenic silver nanoparticles Ag-nps-antibiotics disc diffusion assay

**Fig. 6:**
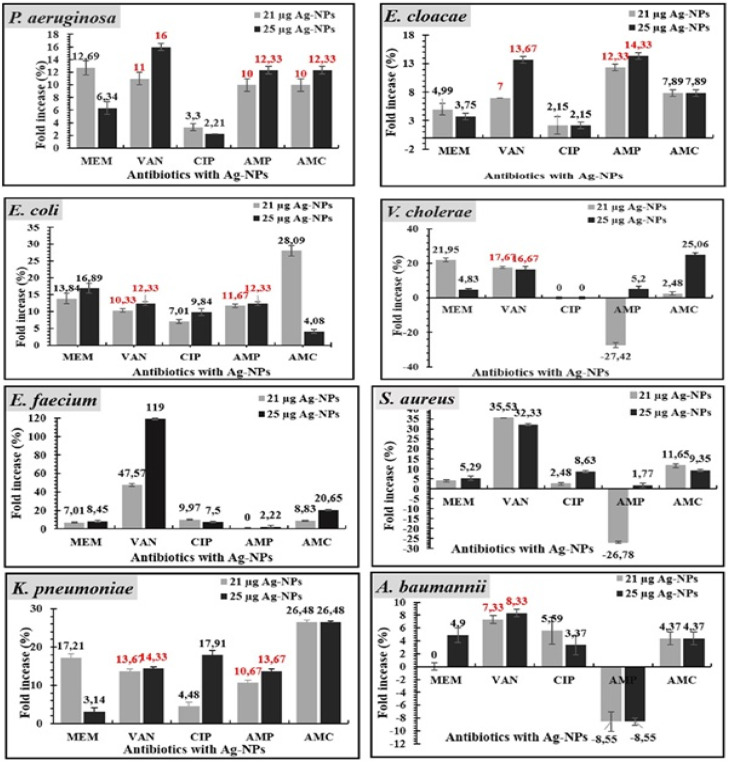
Percentage of fold increase in the area of zones of inhibition of antibiotics in combination with biogenic Ag-NPs. Note: In case of no inhibition zone for antibiotic alone, diameter of inhibition zone of combined treatment was used as percentage value (the values are written in red).

The combined activity of biogenic Ag-NPs with different classes of antibiotics including β-lactam (AMC, AMP, and MEM), glycopeptides (VAN), and quinolones (CIP) was established for all eight bacterial pathogens through disc diffusion assay (Fig. S1 and S2). The average diameter of the zones of inhibition when the antibiotic was combined with either 21 µg or 25 µg of biogenic Ag-NPs and the respective fold increase can be found in Table S1. Fig. 6 is a graphical representation of the percentage-fold increase in the zones of inhibition when antibiotics and biogenic Ag-NPs were combined to inhibit bacterial growth. No percentage fold increase was observed when 21 µg biogenic Ag-NPs were combined with AMP against *E. faecium*, MEM against *A. baumannii* and CIP against *V. cholerae*. Interestingly, a decrease in antibiotic activity was observed when AMP was combined with 21 µg of biogenic Ag-NPs against *V. cholerae* (−27.42%) and *S. aureus* (−26.78%) while both concentrations of biogenic Ag-NPs reduced the effect of AMP against *A. baumannii* as an 8.55%-fold decrease was obtained. Table 2 shows the antibiotic susceptibility profiles for each isolate before and after the combination of biogenic Ag-NPs. Findings show that in several cases the zone of inhibition increased either shifting the susceptibility of the isolate from R to I (*e.g.*, *K. pneumoniae* and *E. cloacae* in the presence of AMC and AMP) or from I to S (*e.g.*, *K pneumoniae* and *E. coli* in the presence of CIP and MEM). For most isolates, the resistance to β-lactams AMC and AMP was maintained even in the presence of biogenic Ag-NPs, albeit an increase in the diameter of the zone of inhibition.^48^

**Table 2:** Antibiotic susceptibility profiles for eight bacterial isolates when treated with antibiotic alone and in the presence of biogenic Ag-NPs^a^

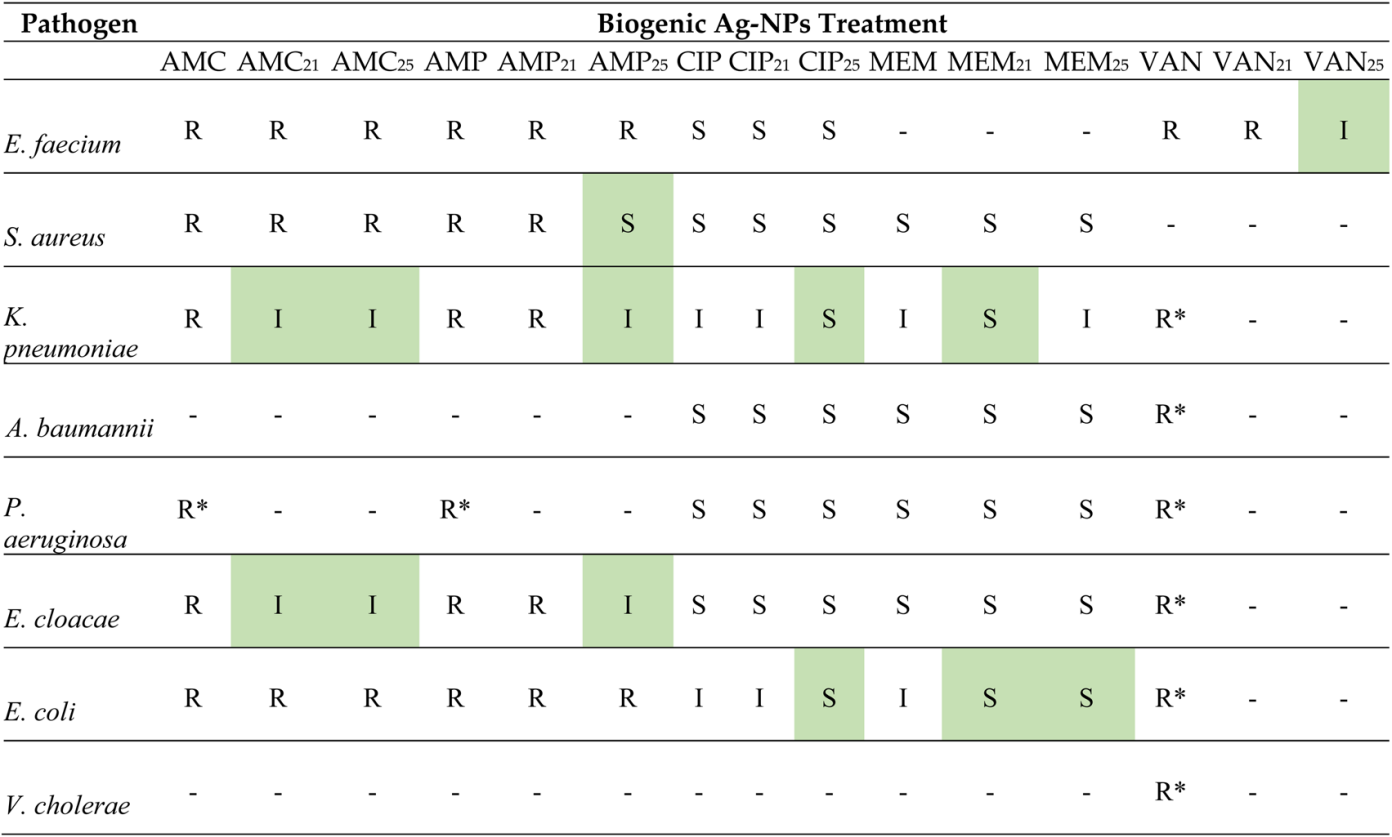

a Bacterial isolates were interpreted as susceptible (S), intermediate (I), or resistant (R) according to CLSI M100 Performance Standards for Antimicrobial Susceptibility Testing (2021) (CLSI report 2021). (−) Zone of inhibition was observed but no breakpoint provided. (*) No zone of inhibition was observed. The shaded areas represent treatments where there was a change in antibiotic susceptibility in the presence of Ag-NPs.

Bacterial isolates were interpreted as susceptible (S), intermediate (I), or resistant (R) according to CLSI M100 Performance Standards for Antimicrobial Susceptibility Testing (2021). (−) A zone of inhibition was observed, but no breakpoint was provided. (*) No zone of inhibition was observed. The shaded areas represent treatments where there was a change in antibiotic susceptibility in the presence of biogenic Ag-NPs.

The emergence and reemergence of MDR bacterial pathogens has been a major concern to public health for over a decade.^31,32^ Their increasing resistance to antibiotics has placed great strain on the pharmaceutical industry to produce new or alternative treatments that can be effective against such pathogens. As a result, the present study sought to determine if antibiotic effectivity can be increased in the presence of biogenic Ag-NPs for the treatment of ESKAPE pathogens. Biosynthesis of biogenic Ag-NPs from *E. xiangfangensis* Pb204 was confirmed due to the presence of a characteristic brown colour change, characteristic SPR absorption peak and visual observation of spherical nanoparticles using TEM. The change in the colour of reaction medium depicts the excitation of surface plasmon vibrations in the metal NPs^33,34^ which occurs due to reduction of Ag^+^ to Ag^0^. These findings are similar to those reported in other studies wherein a yellow to brown colour of the reaction medium was formed due to the reduction of Ag^+^ to Ag^0^.^35^ From the UV-Vis spectra, it is evident that the absorption steadily increases in intensity as a function of reaction time. Absorption peaks appear broad with a low intensity when the reaction time is shorter compared to longer reaction times.^33^ The broadening of peaks is indicative of polydispersity of NPs in the medium, and the absence of additional absorption peaks at higher wavelengths is related to spherical or spherical-like shape uniformity of the NPs^22,41^ as confirmed by TEM. Longer reaction time resulted in larger particle size, ranging from 12–40 nm, and a tendency of NPs to form aggregates was observed. The EDS analysis revealed the presence of only metallic elements, primarily silver, without significant signals from non-metallic components, supporting the metallic nature of the synthesized nanoparticles. Although the exact mechanism of silver nanoparticle formation by *E. xiangfangensis* remains to be elucidated, similar biosynthetic pathways have been reported in other Gram-negative bacteria such as *Escherichia coli* and *Pseudomonas aeruginosa*, where enzymatic reduction of silver ions and the involvement of extracellular proteins have been proposed. These comparisons provide a preliminary framework to hypothesize potential mechanisms in *E. xiangfangensis*, which may involve similar reductase activity or secretion of electron-donating biomolecules.^33,42^ This is due to the secretion of some protein components into the medium from the bacterial biomass, which may be the main factor in the reduction of the metal ions to NPs. Consequently, the proteins may also bind to the NPs enhancing the stability of absorption.^43^

Among the MDR bacteria, ESKAPE pathogens are known to exhibit a range of antibiotic resistance mechanisms including the modification of drug targets, the formation of biofilms, expression of efflux pumps, and enzymatic inactivation. Evidence of resistance to β-lactams (AMC and AMP) was further presented in this study for all bacterial strains tested except for A. baumannii and *V. cholerae* (due to the lack of a CLSI breakpoint). Aside from using combination therapy to combat antimicrobial resistance, the use of biogenic Ag-NPs as an alternative treatment is gaining popularity. In this study, zones of inhibition were detected when 21 or 25 µg of biogenic Ag-NPs were used against the tested pathogens. Although there are no references to establish susceptibility profiles, the measured zones were large enough to confirm an effect against all eight bacteria. A larger zone of inhibition was obtained when a higher concentration of Ag-NPs was used, indicating that the action of these nanoparticles is dose-dependent. Similarly, an increase in concentration of Ag-NPs enhanced their inhibitory effect on *E. coli*, *S. aureus*, *P. aeruginosa,* and *K. pneumoniae*.^49^ Several studies have also demonstrated that the bactericidal properties of Ag-NPs depend on the concentration, size, shape and colloidal state of the nanoparticles.^4,50,51^ Interestingly, Ag-NPs in the present study exhibited the best inhibitory effects against the Gram-positive bacteria *E. faecium* as compared to the other seven tested strains. By contrast, previous studies reported that Gram-negative bacteria are most susceptible to Ag-NPs due to their thin peptidoglycan layer within the cell wall making it easy for Ag-NPs to penetrate.^19,49,52,53^ However, Ag-NPs are reported to have a variable effect on different microorganisms due to multi-mode of action.^54–56^ Multiple mechanisms of Ag-NPs have been suggested to explain their antibacterial mode of action, including the generation of reactive oxygen species (ROS), the inactivation of enzymes, the disruption of cell structure, DNA condensation, and the inactivation of DNA replication. While these actions can sometimes be detrimental to the host, at low concentrations, Ag-NPs are reported to exhibit selective toxicity towards pathogens, making them favorable antimicrobial agents.

The enhanced activity of antibiotics combined with Ag-NPs is explained by the simultaneous effect of silver on various bacterial structures and metabolic processes. Silver nanoparticles can inhibit production of enzymes responsible for bacterial MDR, or even directly inhibit the enzymatic process of antibiotic hydrolysis. In addition, Ag-NPs can act as a drug carrier by transporting antibiotics to the cell surface, wherein they interact with the sulfur-bound proteins, resulting in increased cellular membrane permeability. This allows antibiotic infiltration into the cell.^57,58^

Vancomycin in action alone was inactive against all Gram-negative bacteria; *K. pneumonia*, *A. baumannii*, *P. aeruginosa*, *E. cloacae*, *V. cholerae,* and *E. coli* as it is unable to penetrate the outer bacterial membrane. However, the highest percentage of fold increase in this study was recorded for VAN in the presence of biogenic Ag-NPs against Gram-positive (*E. faecium*) as compared to quinolone and β-lactam antibiotics. Similar results wherein VAN was found to have the highest overall synergistic activity in combination with biogenic Ag-NPs for compared with all other tested antibiotics including AMP, CIP and AMX has been reported.^59^ Our results further correlate with other studies that reported susceptibility of bacterial pathogens, *P. aeruginosa*, *K. pneumoniae*, *E. coli,* and *S. aureus* to antibacterial effects of biogenic Ag-NPs alone and in combination.^60,61^ These findings suggested that when VAN is used in combination with biogenic Ag-NPs, it is effective against *E. faecium*.

Silver nanoparticles in this study restored the bactericidal activity of β-lactam antibiotics that were ineffective against certain test strains, such as AMC against *P. aeruginosa* and AMP against *K. pneumonia*, *A. baumannii*, *E. cloacae,* and *P. aeruginosa*. Other studies have also reported on the restoration of AMP's growth inhibition when combined with Ag-NPs in pathogens like *K. pneumoniae*^62^ and *E. coli*.^63^ The synergistic response is likely due to an increase in the concentration of antibiotics at the site of the bacterium, antibiotic interaction, and the ability to facilitate the binding of antibiotics to bacteria.^64^ It is proposed that the Ag-NPs destabilize the bacterial cell membrane, promote antibiotic internalization in the cell, and, in parallel, the bactericidal activity (Vazquez-Munoz *et al.*, 2019).^63^ If bacteria resist the antibacterial action of either Ag-NPs or antibiotics, then the combined action of antibiotics and Ag-NPs combination may disrupt the bacterial cell wall and inhibit the growth of bacteria by increasing ROS generation with either agent alone.^65^

From all the antibiotics tested, all test strains showed the highest sensitivity to CIP when in treatment alone. Intermediate susceptibility was recorded for *K. pneumoniae* and *E. cloacae* but upon combination with Ag-NPs, the activity of CIP was increased albeit by a small percentage fold increase in zone of inhibition. The fold increase was also the lowest recorded increase compared to other antibiotics. Therefore, ESKAPE pathogens still exhibit good susceptibility to CIP, and combination with Ag-NPs may be unnecessary.

## Conclusions

Biogenic Ag-NPs inhibit the growth of bacterial pathogens including ESKAPE pathogens in a dose-dependent manner. Silver nanoparticles also enhanced the bactericidal activity of some antibiotics and restored the bactericidal activity of inactive antibiotics against the tested strains. Overall, combination treatment using antibiotics and biogenic Ag-NPs offers a more effective solution to antimicrobial resistance than either treatment alone. However, there is a need to determine the *in vivo* activity of these biogenic Ag-NPs on both pathogens and the host (either human or animal cells) before their use as therapeutic agents to combat human and animal pathogens.

## Author contributions

PSS: methodology, data curation, software investigation, writing – original draft. RM: methodology, writing – original draft, review and editing, data analysis, formal analysis, software investigation, validation. Kulsum Kondiah: Conceptualization, writing – review and editing, supervision, project administration, resources, funding acquisition.

## Conflicts of interest

The authors declare no conflict of interest.

## Supplementary Material

NA-008-D5NA00787A-s001

## Data Availability

The data supporting this article have been included as part of the supplementary information (SI). Supplementary information: representing the observed results of all disc diffusion assays and measured zones of inhibition. See DOI: https://doi.org/10.1039/d5na00787a.
