# Supplementary material for: Novel biogenic silver nanoparticles produced by Enterobacter xiangfangensis Pb204 reinstate the activity of specific antibiotics against resistant ESKAPE pathogens
Source: Nanoscale Adv. 2025 Dec 15;8(2):701–11. doi: 10.1039/d5na00787a (PMC12703627; doi:10.1039/d5na00787a)
Supplement: NA-008-D5NA00787A-s001 [file NA-008-D5NA00787A-s001.pdf]

Supplementary Information (SI) for Nanoscale Advances.
This journal is © The Royal Society of Chemistry 2025

Novel Biogenic Silver nanoparticles produced by *Enterobacter xiangfangensis* Pb204 reinstate the activity of specific antibiotics against resistant ESKAPE pathogens.

Supplementary information

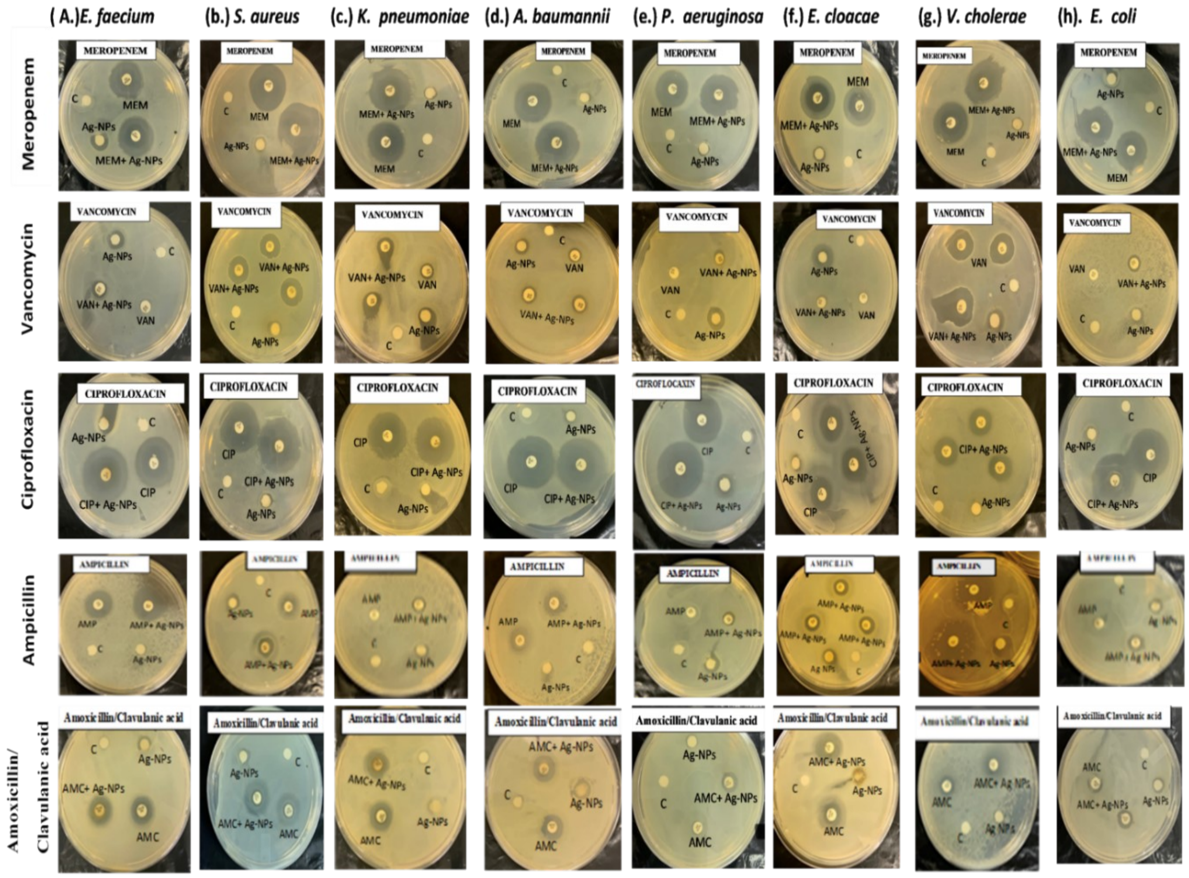

Figure S1. Antimicrobial activity of biogenic Ag-NPs (21 μg) in combination with antibiotics against eight MDR pathogens including ESKAPE pathogens; (A) E. faecium, (B) S. aureus, (C) K. pneumoniae, (D) A. baumannii, (E) P. aeruginosa, (F) E. cloacae (G) V. cholerae, and (H) E. coli. Note: AMP=Ampicillin, CIP=Ciprofloxacin, AMC=Amoxicillin, MEM=Meropenem, VAN=Vancomycin, Ag-NPs = Biogenic silver nanoparticles, and C=Control.

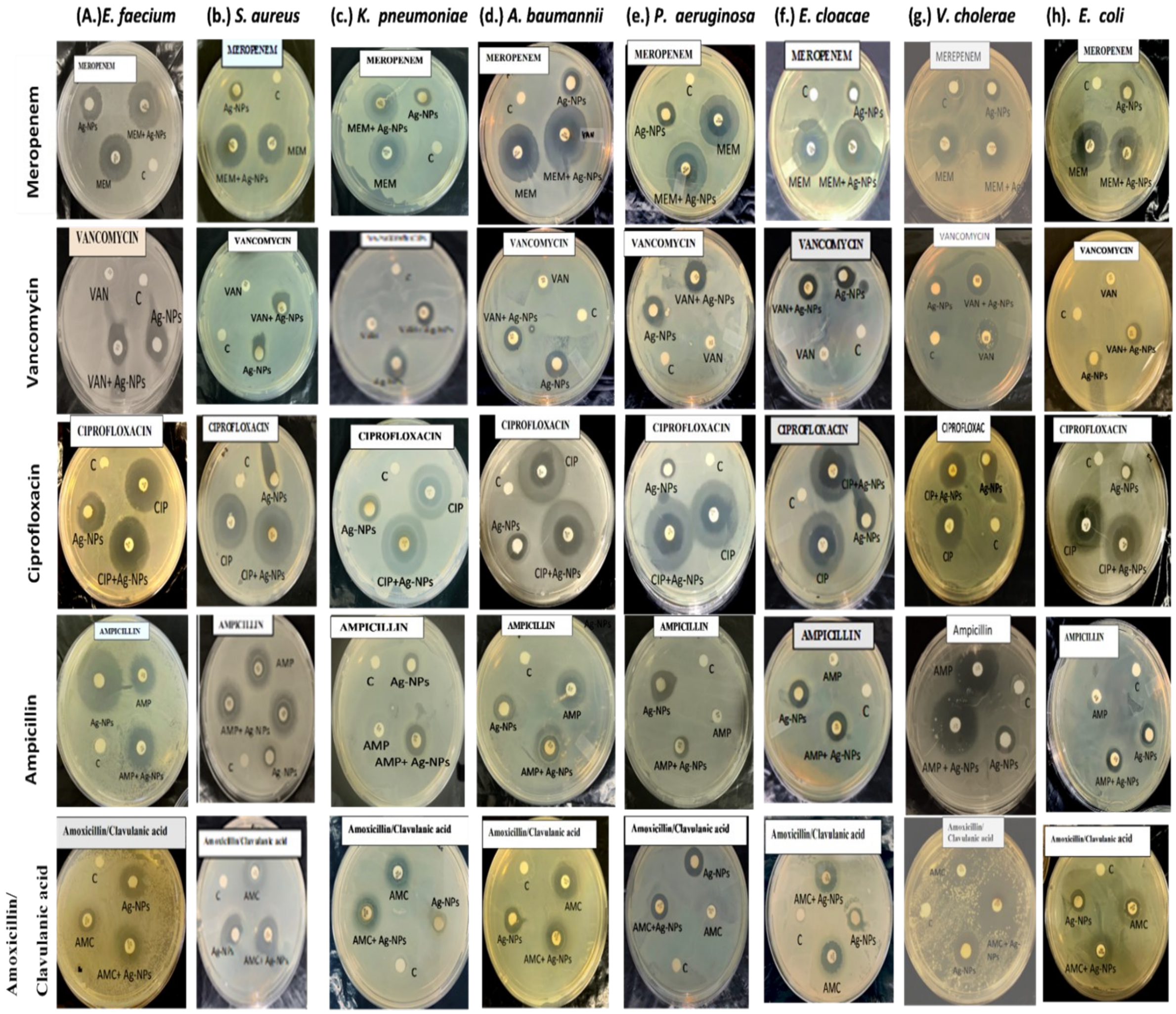

Figure S2. Antimicrobial activity of biogenic Ag-NPs (25 μg) in combination with antibiotics against eight MDR pathogens including ESKAPE pathogens; (A) E. faecium, (B) S. aureus, (C) K. pneumoniae, (D) A. baumannii, (E) P. aeruginosa, (F) E. cloacae (G) V. cholerae, and (H) E. coli. Note: AMP=Ampicillin, CIP=Ciprofloxacin, AMC=Amoxicillin, MEM=Meropenem, VAN=Vancomycin, Ag-NPs =Silver nanoparticles, and C=Control.

Table S1. Inhibition zones for eight bacterial isolates when antibiotic is combined with biogenic Ag-NPs. Data is demonstrated as mean ± S.D. (n=3).

Combined treatment	Treatment																							
	E. faecium			*S. aureus*			*K. pneumoniae*			*A. baumannii*			*P. aeruginosa*			*E. cloacae*			*E. coli*			*V. cholerae*		
	A[1]	B[2]	C[3]	A	B	C	A	B	C	A	B	C	A	B	C	A	B	C	A	B	C	A	B	C
AMC$_{21}$	11.3	12.3	0.18	14.3	16	0.25	11.3	14.3	0.60	15.3	16	0.09	-	10	-	12.7	13.7	0.16	8.3	10.7	0.66	13.3	13	-0.05
AMC$_{25}$	11.3	13.7	0.47	14.3	15.7	0.21	11.3	14.3	0.60	15.3	16	0.09	-	13.3	-	12.7	13.7	0.16	8.3	8.7	0.10	13.3	16.7	0.58
AMP$_{21}$	15.3	15.3	0.00	18.7	13.7	-0.46	-	10.7	-	15.7	14.3	-0.17	-	10	-	-	12.3	-	-	11.7	-	22.5	16.3	-0.48
AMP$_{25}$	15.3	15.7	0.05	18.7	19	0.03	-	13.7	-	15.7	14.3	-0.17	-	12.3	-	-	14.3	-	-	12.3	-	22.5	23.7	0.11
CIP$_{21}$	26.7	29.3	0.20	27	27.7	0.05	22.3	23.3	0.09	29.7	31.3	0.11	30.3	31.3	0.07	30.7	31.3	0.04	23.7	25.3	0.14	15.7	15.7	0.00
CIP$_{25}$	26.7	28.7	0.16	27	29.3	0.18	22.3	26.3	0.39	29.7	30.7	0.07	30.3	31	0.05	30.7	31.7	0.07	23.7	26	0.20	15.7	15.7	0.00
MEM$_{21}$	23.7	25.3	0.14	25.3	26.3	0.08	21.3	25	0.38	27.3	27.3	0.00	26.3	29.7	0. 28	26.7	28	0.10	21.7	24.7	0.30	13.7	16.7	0.49
MEM$_{25}$	23.7	25.7	0.18	25.3	26.7	0.11	21.3	22	0.07	27.3	28.7	0.11	26.3	28	0.13	26.7	27.7	0.08	21.7	25.3	0.36	13.7	14.3	0.09
VAN$_{21}$	7	10.3	1.17	10.3	14	0.85	-	14.3	-	-	7.3	-	-	11	-	-	7	-	-	10.3	-	-	17.7	-
VAN$_{25}$	7	15.3	3.78	10.3	13.7	0.77	-	13.7	-	-	8.3	-	-	16	-	-	13.7	-	-	12.3	-	-	16.7	-

Note: The fold increase was calculated using the formula: Fold increase = $(B^2-A^2)/A^2$, wherein A and B is the diameter of zone of inhibition (mm) obtained for antibiotic alone and antibiotic in combination with biogenic Ag-NPs respectively. Where there was no inhibitory effect for antibiotic in treatment alone, diameter of the disc (6 mm) was used for calculations.

- A- diameter of zone of inhibition (mm) obtained for antibiotic alone
- B- the diameter of zone of inhibition (mm) obtained for antibiotics in combination with biogenic Ag-NPs respectively.
- C- the increase in fold area
